# Dissolution of Microparticles of Cadmium, Lead and Thallium in Water

**DOI:** 10.3390/toxics13110904

**Published:** 2025-10-22

**Authors:** Gennadii L. Bykov, Boris G. Ershov

**Affiliations:** Frumkin Institute of Physical Chemistry and Electrochemistry, Russian Academy of Sciences, Leninsky Pr. 31-4, Moscow 119071, Russia

**Keywords:** PM_2.5_, cadmium, lead, thallium, microparticles, oxidation, dissolution, sedimentation, hazard

## Abstract

Anthropogenic activity seriously damages the environment. Cadmium, lead, and thallium are toxic elements that are especially hazardous for nature. In polluted air, they are present in the form of microparticles 2–3 μm in size and belong to the PM_2.5_ fraction. Such particles can be transported over long distances, penetrate into water and dissolve, and then enter the food chain. This poses a severe threat to human and animal health due to the bioaccumulation of metals. Therefore, it is important to study the properties of toxic metals of this size. In this work, we developed a radiation–chemical method for obtaining microparticles of cadmium, lead, and thallium corresponding to the PM_2.5_ fraction and studied their properties in aqueous solutions. In the absence of oxygen, the metals do not dissolve. Over time, they agglomerate and settle. When exposed to air, the particles quickly dissolve in water, usually within a few minutes. This process involves the disappearance of small particles and a decrease in the size of larger ones. The rate of dissolution increases in the Pb-Cd-Tl series. Cadmium dissolves approximately 4–5 times faster than lead, and thallium more than 10 times faster. Acidification of water accelerates this process. Studying the properties of microparticles of heavy metals is important for assessing their migration in the environment, health risks, and developing methods for preventing pollution.

## 1. Introduction

Heavy metals are hazardous environmental pollutants. Cadmium, lead, and thallium are the most hazardous to human health and the environment due to their toxicity, cancer-causing potential, and accumulation in food chains [1,2,3,4,5]. The main sources of their entry into the environment are associated with the entry of nano- and microparticles into the atmosphere during the development of ore deposits, the operation of metallurgical enterprises, as a result of industrial and automobile emissions, the combustion of coal and oil, and many other ways. According to [1,3,6,7,8], particles up to 2–3 µm in size predominate in the atmosphere. Elevated levels of heavy metals, including Cd, Pb, and Tl PM_2.5_ fraction, were detected in northern and southern metropolitan areas of China [1]. A similar situation is observed in other regions of the world [3,6,7,8]. In particular, lead microparticles are present in the atmosphere of cities and industrial areas. For example, lead nanoparticles ranging in size from 0.05 to 15 μm were detected in Helsinki, with an average concentration of 0.13 ng/m^3^ [7]. Lead micro- and nanoparticles were also detected in the air of Amman, Jordan [8]. The average mass concentration of the PM_2.5_ fraction was 18 μg/m^3^. Solid particles of this size are the most hazardous to living organisms. According to WHO [9], in most areas of Europe, PM_2.5_, i.e., particles up to about 2.5 µm in size, make up 50–70% of PM_10_ particles. It is important to note that 3% of deaths from cardiovascular and respiratory diseases and 5% of deaths from lung cancer are associated with PM_2.5_. Toxic metals play a key role in this. The hazard of microparticles of toxic metals is associated not only and not so much with their small size and the permeability of membranes of organisms for them. A significantly greater hazard is associated with their toxicity. Due to their small size, they can remain suspended in the atmosphere for a long time and, thus, travel long distances. Water (sea, natural springs, reservoirs, etc.) is a virtually obligatory intermediate link in their subsequent distribution in the natural environment. It is in water that metals oxidize and, in the form of ions and compounds, then enter the soil and organisms. Therefore, it is important to study the properties of cadmium, lead, and thallium microparticles and their solubility in water. This is difficult due to their high chemical activity, the need to develop special methods for obtaining particles of the desired size, and protective measures when working with them. Earlier, we developed a radiation-chemical method for obtaining nano- and microparticles of cadmium [10,11]. We found that aqueous dispersions of cadmium microparticles of 2–3 μm in size are unstable. They quickly oxidize in air and dissolve in water of various compositions and origins.

This work continues the study of microparticles of toxic metals. Our goal was to compare how hazardous cadmium, lead, and thallium dissolve in water and how this process is affected by various environmental factors, such as air, pH, and various ions. For the first time, we have established the mechanism by which microparticles of metals convert to soluble forms upon interaction with water. It was found that small particle sizes significantly accelerate their dissolution, and dissolution rates increase in the metal series in the Pb-Cd-Tl sequence. These results are important to consider when developing strategies to address environmental issues and prevent the spread of toxic metals into the environment.

## 2. Materials and Methods

### 2.1. Chemicals and Reagents

The chemicals used in the study were of high analytical quality and were used without additional purification. The following chemicals were used: 3CdSO_4_·8H_2_O (extra pure), Pb(ClO_4_)_2_ (Acros Organics, Geel, Belgium), Tl_2_SO_4_ (extra pure), 2-propanol (CH_3_)_2_CHOH (99.8%, EKOS-1, Kupavna, Russia), hydrochloric acid HCl (extra pure, EKOS-1, Russia), NaOH (99.9%, Khimmed, Moscow, Russia), and Na_2_CO_3_ (99.9%, Khimmed, Russia). The solutions were prepared using triple-distilled water.

### 2.2. Preparation of Metal Dispersions

The following methods and installations were used in the experiments. The solutions were irradiated using an LINS-02-500 linear accelerator (RadiaBeam Technologies, Santa Monica, CA, USA). The monochromatic radiation energy was 2 MeV, the pulse length was 4 μs, and the pulse frequency was 50 Hz. The absorbed dose rate was measured with an iron sulfate dosimeter. The absorbed dose varied from 10 to 100 kGy.

Solutions of cadmium, lead, or thallium salts, which also contained propan-2-ol, were irradiated with accelerated electrons in a closed glass vessel. The irradiation was performed in 30 cm^3^ glass test tubes of 3 × 9.5 cm size and in flat cells 1 cm thick and 4.5 cm in diameter with a volume of 10 mL. Prior to irradiation, the solutions were degassed by freezing–pumping–thawing. The vessel carried a sidearm with an optical quartz cuvette. Another sidearm carried a septum. Optical spectra or dynamic light scattering could therefore be measured, and substances could be added via a syringe without exposure of the solutions to air.

### 2.3. Study of Characteristics of Metal Particles

The UV spectra were recorded with a Cary 100 spectrophotometer (Agilent Technologies, Santa Clara, CA, USA) using 1 and 0.5 cm thick quartz cells. The hydrodynamic size of the resulting metal nanoparticles was determined with a Delsa Nano C device (Beckman Coulter, Indianapolis, IN, USA) by photon correlation spectroscopy using a laser with a wavelength of 658 nm; the scattering angle was 160°.

The photographs of the surface of deposited metals and colloidal particles were taken with a Revelation III (model R3M-BN4A-DAL3) binocular microscope (LW Scientific, Lawrenceville, GA, USA). The images were processed using the ScopePhoto 3.0 and ImageJ with 64-bit Java 8 (ensemble OC 190.45.4780, https://imagej.net/ij/download.html, accessed on 16 October 2025) programs. After irradiation of the deaerated solutions, the metal microparticles were examined using microscopy. They were first examined at the bottom of the optical cell without air access. Then air was passed through to evaluate the changes. The oxidative dissolution of the particles was analyzed by applying drops of the dispersion to glass. The entire preparation process, including the application of the drops, took about one minute before the measurements began.

The SEM images were taken with a KYKY-EM6900 scanning electron microscope (KYKY Technology Co., Ltd., Beijing, China) equipped with a thermoemission tungsten cathode. The device allowed taking SEM images and performing elemental mapping by energy-dispersive X-ray (EDX) spectroscopy in real time. The resolution was 3 nm at 30 kV. The accelerating voltage was from 0 to 30 kV.

The reduction in metal ions (Cd^2+^, Pb^2+^, or Tl^+^) occurs by the radicals that are generated by irradiation of aqueous solutions. The radiolysis leads to the uniform generation of radical and ionic species throughout the volume: hydrated electrons (e^−^_aq_), hydrogen atoms (H^•^), and hydroxyl radicals (^•^OH); their radiation–chemical yields are *G*(e_aq_^−^) = 0.27, *G*(H^•^) = 0.06, and *G*(^•^OH) = 0.28 µmol J^−1^ [12]. The ^•^OH radical and H^•^ atom enter into the dehydrogenation reaction with propan-2-ol:

^•^OH (H^•^) + (CH_3_)_2_CHOH → H_2_O (H_2_) + (CH_3_)_2_^•^COH (1)

In these conditions, only two reducing species are formed: e_aq_^−^ (E^0^ = −2.87 V) and (CH_3_)_2_^•^COH (E^0^ = −1.34 V), respectively [13,14]. In this case, the action of accelerated electrons on an aqueous solution containing metal ions initiates their reduction to form a metal [15]. Comparison of the reduction potentials of these radical species with the electrode potentials of the metals under study (−0.403 V, −0.126 V, and −0.336 V for pairs Cd^2+^/Cd^0^, Pb^2+^/Pb^0^, and Tl^+^/Tl^0^, respectively) shows that the reduction in their ions in water is thermodynamically possible. The results of the studies confirm this. The total concentration of reducing species in this case formed at an absorbed dose of 1 kGy is approximately 6 × 10^−4^ mol L^−1^ kGy^−1^. Schematically, the reduction process using cadmium as an example can be expressed by the following reactions:

Cd^2+^ + 2e^−^_aq_ → Cd^0^ (2)

Cd^2+^ + 2(CH_3_)_2_^•^COH → Cd^0^ + 2(CH_3_)_2_CO + 2H^+^ (3)

nCd^0^ → Cd_n_ (4)

As a result, aqueous dispersions of the metals being studied are formed. According to reactions (2) and (3), the concentrations of Cd^0^ and Pb^0^ atoms formed at an absorbed dose of 1 kGy should be expected to be ~3 × 10^−4^ mol L^−1^ kGy^−1^, and for Tl^0^ atoms the concentration should be twice as high.

The studies showed that the radiation–chemical method is suitable for obtaining aqueous dispersions of cadmium, lead, and thallium. The main fraction of particles corresponded to PM_2.5_—that is, did not exceed 2.5 μm in diameter. Such particles predominate in the air of large cities [1,3,6,7,8,9].

## 3. Results

### 3.1. Formation and Characteristics of the Metal Dispersions in Water

**Figure 1:**
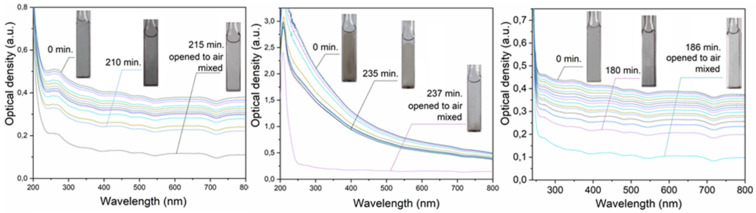
Evolution of the optical spectra of the deaerated solutions of Cd, Pb, and Tl salts (0.01 mol L^−1^), containing propan-2-ol (0.1 mol L^−1^), over time after irradiation. The concentration of metal atoms is approximately equal to ~2 × 10^−3^ mol L^−1^.

Deaerated aqueous solutions of Cd, Pb, and Tl salts containing propan-2-ol acquire a gray-brown color after irradiation, which is caused by the formed metal particles. Figure 1 illustrates the optical spectra of aqueous dispersions. In the case of cadmium and thallium, a spectrum without pronounced maxima in the UV and Vis regions is observed. Such a flat optical spectrum is caused by the scattering and reflection of light by the formed metal particles, the size of which is comparable (d ≥ 0.1λ) to or exceeds the wavelength of light (the so-called diffuse transmittance) [16]. In the case of lead, a spectrum increasing in the UV region is observed, caused by the superposition of an additional absorption band of plasmons in this metal on the diffuse transmittance spectrum. It is known that lead nanoparticles have an absorption band at 218 nm with a molar absorption coefficient of 3.2 × 10^4^ mol L^−1^ cm^−1^ induced by a surface plasmon oscillation [17].

The optical density of the plasmon band (Figure 1) and the molar absorption coefficient allows us to calculate the concentration of Pb^0^ atoms in the form of nanoparticles. This is approximately 1–2% of the total metal concentration in the dispersion. With an increase in the concentration of Pb^0^ atoms, the relative intensity of the plasmon band of nanoparticles decreases due to their aggregation. The addition of CO_3_^2−^ and SO_4_^2−^ anions to an aqueous lead dispersion immediately leads to the disappearance of the plasmon band. This occurs due to the sorption of anions, which neutralizes the charge of the metal core of colloidal particles. The stabilizing double electric layer is destroyed, and the nanoparticles lose stability and aggregate. The spectrum becomes similar to that observed for cadmium and thallium dispersions.

**Figure 2:**
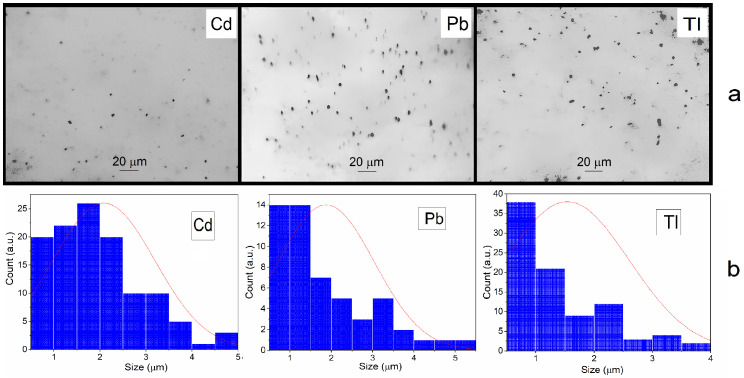
Micrographs of metal particles at the bottom of the cell taken immediately after irradiation of deaerated solutions of Cd, Pb, and Tl salts (0.01 mol L^−1^), containing propan-2-ol (0.1 mol L^−1^) (**a**) and particle size distribution (**b**). Magnification ×1000. Average particle sizes are Cd 2.3 ± 0.3 μm, Pb 2.0 ± 0.1 μm, and Tl 1.8 ± 0.2 μm.

The production of metal microparticles was studied at doses ranging from 10 to 100 kGy. As the dose increased, the metal concentration increased, while the size remained within 2–3 μm. The optimal dose for PM_2.5_ particle studies was chosen to be 10 kGy at a metal concentration of ~2 × 10^−3^ mol L^−1^. The size of the metal particles formed in deaerated solutions after irradiation is 2.3 ± 0.3 μm, 2.0 ± 0.1 μm, and 1.8 ± 0.2 μm for Cd, Pb, and Tl dispersions, respectively (Figure 2). The mean mass concentration of the PM_2.5_ fraction, particulate matter with a diameter of 2.5 μm or less, is 80% or more. That is, by their size, they correspond to the main fraction of toxic metals present in the polluted air environment of megacities [1,2,3,6,7,8,9]. The formation of metal microparticles is accompanied by a decrease in the pH of solutions, i.e., their acidification. This is caused by the accumulation of negatively charged electrons by metals and the formation of positive H^+^ ions in the solution. For example, the pH of a cadmium salt solution is 6.6, and after irradiation at a dose of 10–20 kGy, this value decreased to 5.0.

**Figure 3:**
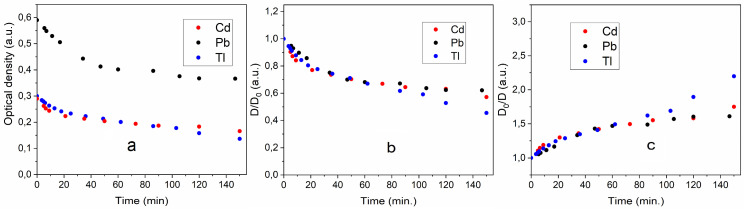
Change in the optical density of Cd, Pb, and Tl dispersions in coordinates D_t_ (**a**), D_t_/D_0_ (**b**), and D_0_/D_t_ (**c**) over time (according to data in Figure 1).

Metal dispersions are unstable (Figure 1). Over time, the particles stick together and settle. Sedimentation makes the solutions more transparent. Figure 3 shows how the optical density of the dispersions decreases over time. There are two stages of optical density reduction. The first occurs quickly, within 50–70 min. The second lasts for several hours. These two stages are clearly revealed if the dependence of the change in optical density on time is expressed in coordinates D_t_/D_0_ and D_0_/D_t_. The D_t_/D_0_ coordinates reflect the change in turbidity, and the D_0_/D_t_ coordinates reflect the change in transparency. These dependencies are shown in Figure 3b,c.

It can be seen that the change in optical density over time in the fast region is the same for dispersions of cadmium, lead, and thallium. It can be assumed that the change in optical density here is due to the agglomeration of the formed metal particles. The slow sections are approximately the same for lead and thallium. However, for cadmium, the turbidity decreases more slowly, and, accordingly, the dispersion also becomes clearer more slowly. Complete clarification of the dispersion and the release of the metal into the sediment takes almost a day. Dispersions of all the metals studied quickly dissolve when exposed to air (Figure 1).

Sedimentation of metal microparticles under the action of gravity occurs in the laminar region, where Stokes’ law applies [18]. The settling velocity of cadmium particles of 2–3 μm in size, as measured by the change in suspension turbidity, is about 1.3 × 10^−5^ m s^−1^ [11]. This value agrees well with the calculated value for spherical particles—6.7 × 10^−6^ m s^−1^. Considering that the calculations were carried out for a monodisperse system, the results of the experiments and theoretical calculations can be considered satisfactory.

In our studies, the characteristics of cadmium, lead, and thallium solutions were the same and did not change, and the particle sizes were also approximately the same. According to Stokes’ law [18], differences in the settling velocities of microparticles of these metals can be explained by the difference in their specific masses. For cadmium, lead, and thallium, they are 8.7 g cm^−3^, 11.3 g cm^−3^, and 11.8 g cm^−3^ respectively [19]. The mass ratio of lead and thallium to cadmium suggests that their sedimentation rate should be 35–40% higher than that of cadmium. This roughly corresponds to the changes in the optical densities of solutions of these metals over time (Figure 3).

Increasing the concentration of metals increases the particle size and accelerates their sedimentation [10,11]. However, in this paper we will focus on the PM_2.5_ particle fraction. They constitute a significant part of the atmospheric pollution with toxic metals. The study showed that Cd, Pb, and Tl microparticles in deaerated water are stable. Mixing the sediment returns the system to a dispersed state, and subsequent storage is accompanied by sediment formation. Microscopy revealed particle aggregation over time. Microparticles are not dissolved and retain their size and shape during long-term storage without air. However, they quickly dissolve in the presence of air.

### 3.2. Oxidative Dissolution of the Metal Microparticles in Water

**Figure 4:**
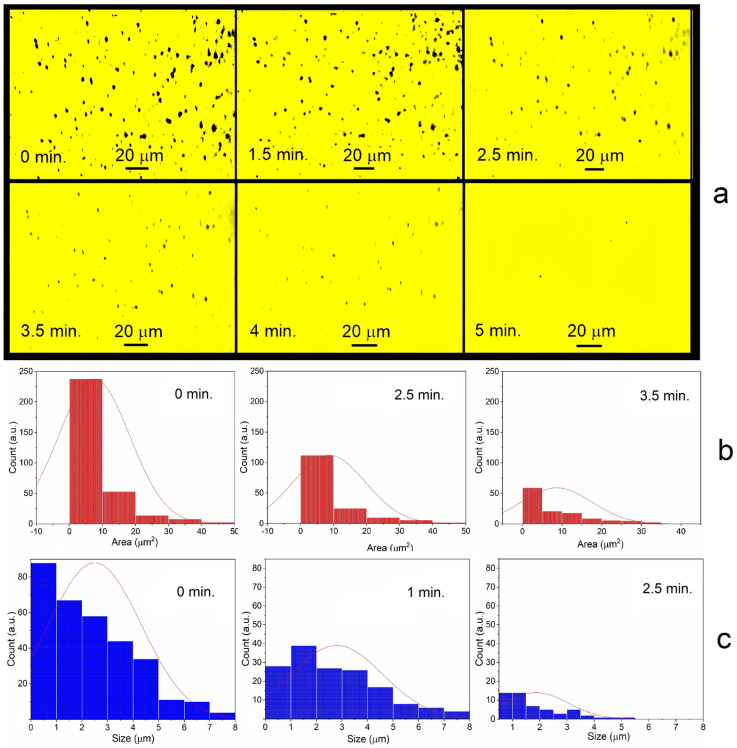
Micrographs of Pb microparticles on the glass surface (**a**), changes in their area (**b**), and size (**c**) over time. Magnification ×1000. Average particle sizes are 2.5 ± 0.1 μm (0 min.), 2.8 ± 0.07 μm (1 min.), and 2.0 ± 0.05 μm (2.5 min.).

As already mentioned, cloudy-gray aqueous dispersions of cadmium, lead, and thallium become transparent after a few minutes of saturation with air and mixing (Figure 1). This is due to the oxidative dissolution of microparticles. Figure 4 shows this process in dynamics on the glass surface in several drops of lead dispersion. The sizes and areas of microparticles were recorded by photomicroscopy along their contours. Deaerated drops of the solution were applied to a glass plate in air. The first measurement was taken after about one minute.

Figure 4 shows that the metal microparticles disappear and decrease in size in about 4–5 min. Small particles disappear first, while large ones decrease in size. The average particle diameter is 2.3 ± 0.3 μm. The particle size distribution changes noticeably only at the last stage of dissolution. This is probably due to the fact that the disappearance of small particles is compensated by the appearance of new ones of the same size due to the dissolution of large particles. It was found that acidifying a deaerated solution of lead microparticles had virtually no effect on their stability. However, upon exposure to air and shaking, the microparticles dissolved significantly faster (within 1 min). This indicates that the presence of H^+^ ions accelerates metal dissolution.

After dissolution of the microparticles, the original optical absorption spectrum of the solutions and their pH values are restored. This indicates that the products of oxidative dissolution have the same chemical composition and ionic forms as the original solutions.

**Figure 5:**
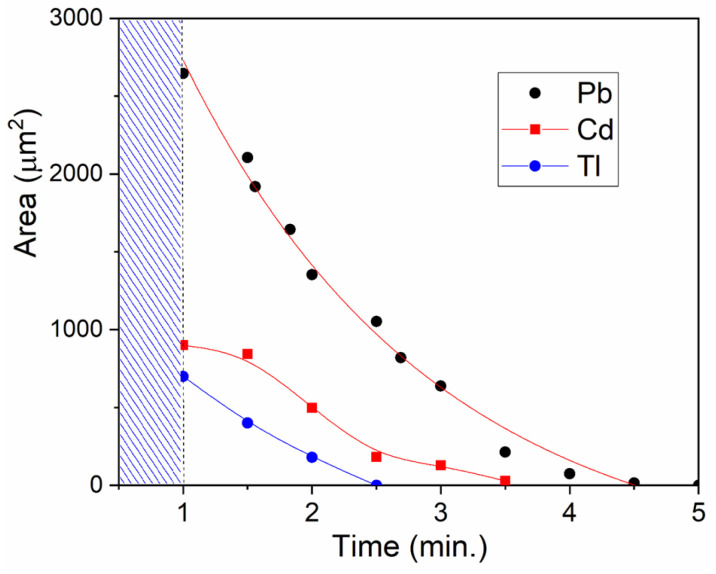
Change in the surface area of microparticles in air over time. The time for preparing the metal dispersion for measurement was 1 min. The shaded area is the period of preparation for measurements.

The dynamics of cadmium and thallium dissolution resemble the process of lead dissolution. First, small particles disappear, then large ones dissolve (Supplementary Materials). However, there is an important difference: the dissolution rate increases in the series lead—cadmium—thallium. The concentrations of metals in deaerated solutions were approximately the same immediately after their preparation (Figure 1). But by the time of the first measurement, the total surface area for cadmium was 3–4 times smaller than for lead, and for thallium, almost 10 times smaller (Figure 5). After one minute of cadmium and thallium dispersions being in the air, their concentrations significantly decreased compared to lead. Dissolution of these metals also occurs more quickly. The subsequent dissolution of lead takes about 5–6 min, cadmium takes 2–3 min, and thallium takes about 1.5 min.

It can be concluded that most of the cadmium and especially thallium microparticles dissolved within one minute of exposure to air, even before the microscopy was started. Microparticles of cadmium, lead, and thallium dissolve so rapidly that traditional methods do not allow us to measure their rate constants. The time required for measurements is comparable to or exceeds the dissolution times of these metals, particularly thallium and cadmium. Therefore, their relative dissolution rates were estimated by measuring the change in the total surface area of the particles in solution on a glass plate using a high-resolution microscope (Figure 4). The program calculated the change in surface area over time. It turned out that the relative dissolution rates are related as 1:4:10 for metals in the Pb-Cd-Tl sequence (average of five measurements, error ±30%).

Natural waters contain a variety of compounds with which dissolved metal ions form new soluble or insoluble products. The chemical and physical state of this product is important for the further distribution and accumulation of toxic metals in the biosphere.

**Figure 6:**
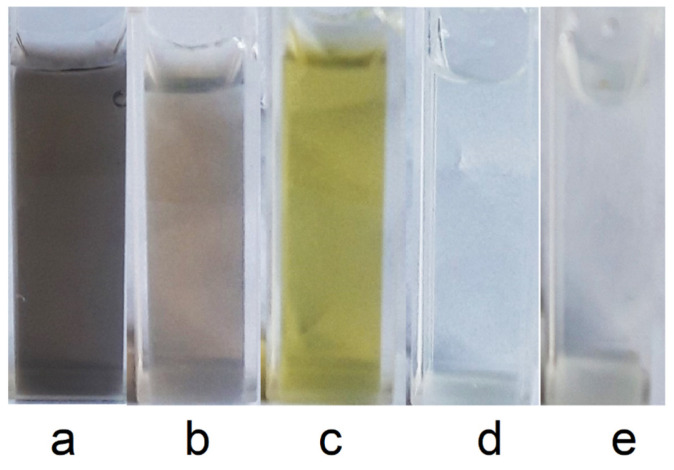
Dispersion of lead microparticles (**a**), and after addition of a solution of Na_2_MoO_4_ (**b**), Na_2_CrO_4_ (**c**), Na_2_CO_3_ (**d**), and K_3_PO_4_ (**e**) salts.

Addition of Na_2_MoO_4_, Na_2_CrO_4_, Na_2_CO_3_, or K_3_PO_4_ salt solutions to a solution of lead microparticles results in their rapid dissolution in air, forming a dispersion of insoluble and colored lead salts (Figure 6).

No direct relationship between the rate of oxidative dissolution of metals and the values of their electrode potentials (−0.403 V, −0.126 V, and −0.336 V for pairs Cd^2+^/Cd^0^, Pb^2+^/Pb^0^, and Tl^+^/Tl^0^, respectively [19]) is found here. The rate is probably affected by the formation of a protective oxide film and its subsequent relatively slow dissolution. Indeed, TlOH is highly soluble (34.3 g in 100 mL of water), while Cd(OH)_2_ is poorly soluble (0.0015 g) and Pb(OH)_2_ is even less soluble (0.00012 g) [19]. It has been found that the acidity of water accelerates the dissolution of metals, while alkalinity, on the contrary, slows down this process.

Dissolution of cadmium, lead, and thallium is activated in the presence of air. The standard reduction potential of the reaction O_2_ + 4H^+^ + 4e^−^ ⇌ 2H_2_O is 1.229 V [19]. The electromotive force of the oxidation reaction of metals ΔE = E(O_2_/H_2_O) − E(M^n+^/M_s_) is 1.632 V for cadmium, 1.355 V for lead, and 1.565 V for thallium, respectively. Such a large EMF favors its active course, i.e., oxidation of metals. During oxidation, metal hydroxides are apparently formed at the initial stage—poorly soluble Cd(OH)_2_ and Pb(OH)_2_. A protective film is formed, hindering the development of the corrosion process of their dissolution. The formation of a protective oxide film is confirmed by data from a study [10,11]. It was shown that the rate of dissolution of cadmium microparticles significantly increases when the material is exposed to air, resulting in the formation of an oxide that protects the metal from corrosion. The role of the acid is that it dissolves the hydroxides and, thus, favors oxidative dissolution. Then the stoichiometry of oxidative dissolution of Cd and Pb can be written as follows:

2Cd + O_2_ + 4H^+^ → 2Cd^2+^ + 2H_2_O (5)

2Pb + O_2_ + 4H^+^ → 2Pb^2+^ + 2H_2_O (6)

In the case of thallium, soluble TlOH does not hinder the corrosion of the metal and therefore its dissolution rate is the highest.

4Tl + O_2_ + 4H^+^ → 4Tl^+^ + 2H_2_O (7)

The oxidative dissolution of cadmium, lead, and thallium, conductive materials, in an electrolyte, which is water, occurs by an electrochemical mechanism. This mechanism underlies the corrosion of metals and their dissolution.

## 4. Conclusions

**Figure 7:**
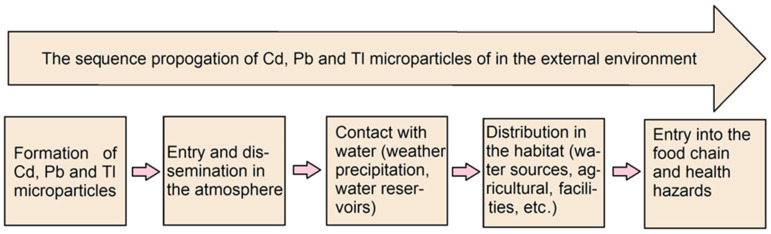
Scheme of formation and distribution of Cd, Pb, and Tl microparticles in the natural environment.

According to the World Health Organization, fine particulate matter (PM_2.5_), regardless of its origin, is harmful to human health and the environment. Particularly hazardous are microparticles of cadmium, lead, and thallium. They are formed during ore mining, coal burning, industrial processes, and vehicle exhaust. These particles spread into the air, negatively impacting health and the environment. Their distribution in the external environment can be expressed in the most general form by the following scheme (Figure 7):

In a polluted atmosphere, ultrafine particles of 2–3 μm and smaller (PM_2.5_) predominate. They pose a severe threat to bioorganisms, as they contain toxic metals. These metals can remain in the air for a long time and be transported over long distances. Water acts as an intermediary, transforming these metals into soluble forms that can spread even further. Obtaining entry into food chains, they accumulate in organisms, causing hazardous diseases. The environmental problem associated with hazardous toxic metals conventionally includes two main components: the dissemination of 2.5 μm microparticles in the atmosphere (1) and the contact with natural waters (2). This research bridges the gap between these two concerns by uncovering the mechanism through which these particles transform into soluble forms. The study showed that in an ultrafine state, cadmium, lead, and thallium have high chemical activity. In the presence of air, these metals dissolve extremely rapidly, often within minutes. The rate of dissolution increases in the series Pb-Cd-Tl. As a result, microparticles of metals transform into ionic form, which poses the greatest danger to the environment and can also accumulate in the soil in the form of poorly soluble compounds. The practical significance of this study, in our opinion, lies in its demonstration of the absence of a barrier preventing the transfer of ultrafine toxic metals from the air into water. The transition to soluble and readily transportable chemical forms occurs, so to speak, instantaneously. Metals quickly dissolve and spread throughout the environment. Therefore, understanding the properties of heavy metal microparticles is crucial for evaluating their migration in the environment, assessing health risks, and developing strategies to prevent pollution. As microparticles move into the environment, their distribution area expands, making containment methods more difficult and expensive. Therefore, the most effective and efficient way to address the problem of ultrafine toxic metals is to develop methods and means to prevent their formation and release into the environment.

## Data Availability

Data is contained within the article or Supplementary Materials.

## Supplementary Materials

The following supporting information can be downloaded at: https://www.mdpi.com/article/10.3390/toxics13110904/s1, Figure S1: Micrographs of Cd microparticles on the glass surface (a), changes in their area (b), and size (c) over time. Magnification ×1000. Average particle sizes 2.2 ± 0.1 μm (0 min.), 2.0 ± 0.06 μm (1 min.), and 1.8 ± 0.05 μm (2.5 min.). Figure S2: Micrographs of Tl microparticles in the volume of liquid (a), changes in their area (b), and size (c) over time. Magnification ×1000. Average particle sizes 2.5 ± 0.1 μm (0 min.), 2.2 ± 0.09 μm (1.5 min.), and 1.7 ± 0.05 μm (2 min.).
