# Supplementary material for: Dissolution of Microparticles of Cadmium, Lead and Thallium in Water
Source: Toxics. 2025 Oct 22;13(11):904. doi: 10.3390/toxics13110904 (PMC12656692; doi:10.3390/toxics13110904)
Supplement: Supplementary file 1 [file toxics-13-00904-s001.zip › Supplementary-Materials.pdf]

Supplementary Materials

Dissolution of Microparticles of Cadmium, Lead and Thallium in Water

*Gennadii L. Bykov * and Boris G. Ershov*

Frumkin Institute of Physical Chemistry and Electrochemistry, Russian Academy of Sciences, Leninsky Pr. 31-4, 119071, Moscow, Russia

*Corresponding author: Gennadii L. Bykov (E-mail: bykov@ipc.rssi.ru)

Number of figures: 2

Figure S1. Micrographs of Cd microparticles on the glass surface (a), changes in their area (b) and size (c) over time. Magnification ×1000. Average particle sizes 2.2±0.1 μm (0 min.); 2.0±0.1 μm (1 min.) and 1.8 ±0.1 μm (2.5 min.).

Figure S2. Micrographs of Tl microparticles in the volume of liquid (a), changes in their area (b) and size (c) over time. Magnification ×1000. Average particle sizes 2.5±0.1 μm (0 min.); 2.2±0.1 μm (1.5 min.) and 1.7±0.1 μm (2 min.).

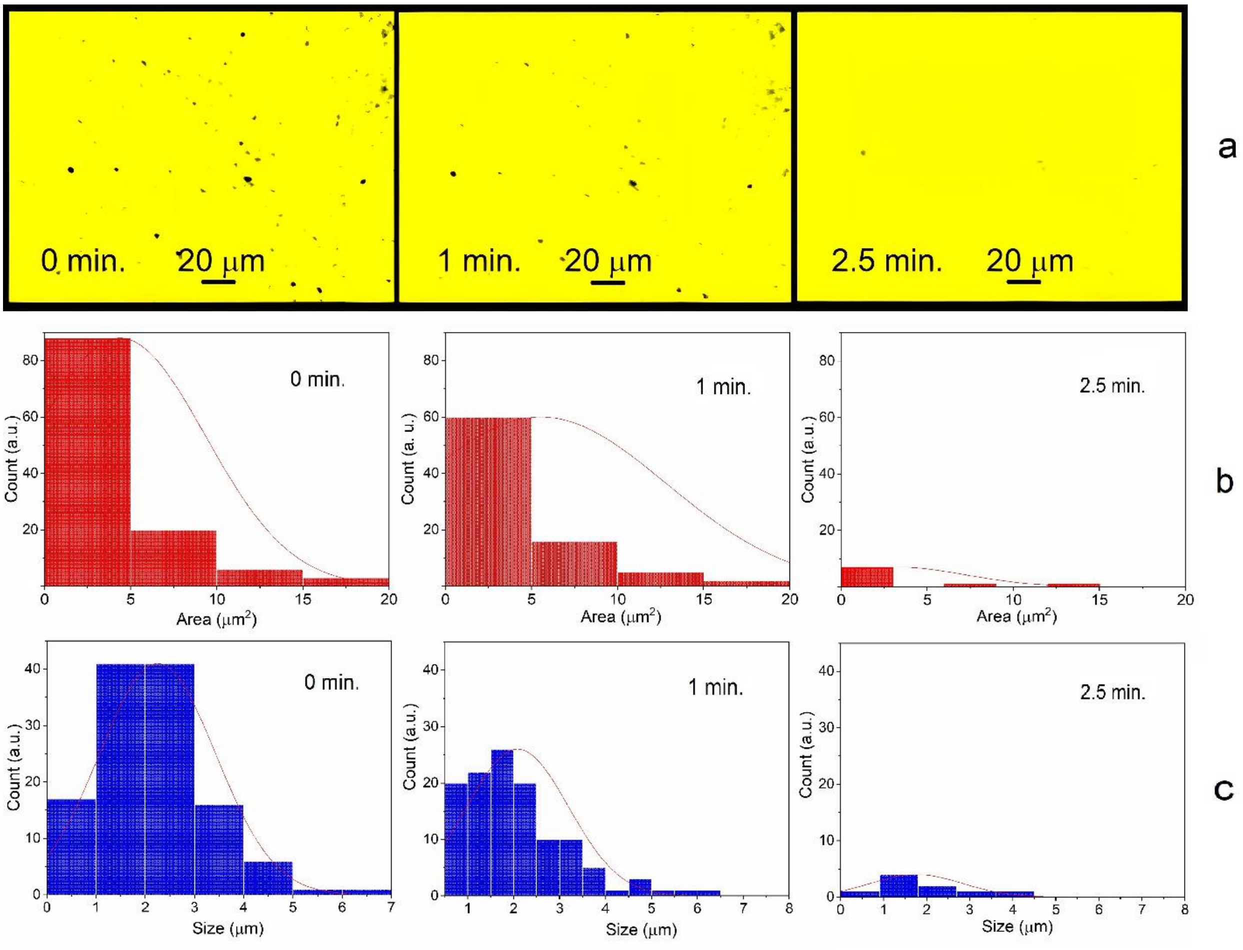

Figure S1. Micrographs of Cd microparticles on the glass surface (a), changes in their area (b) and size (c) over time. Magnification ×1000. Average particle sizes 2.2±0.1 μm (0 min.); 2.0±0.1 μm (1 min.) and 1.8±0.1 μm (2.5 min.).

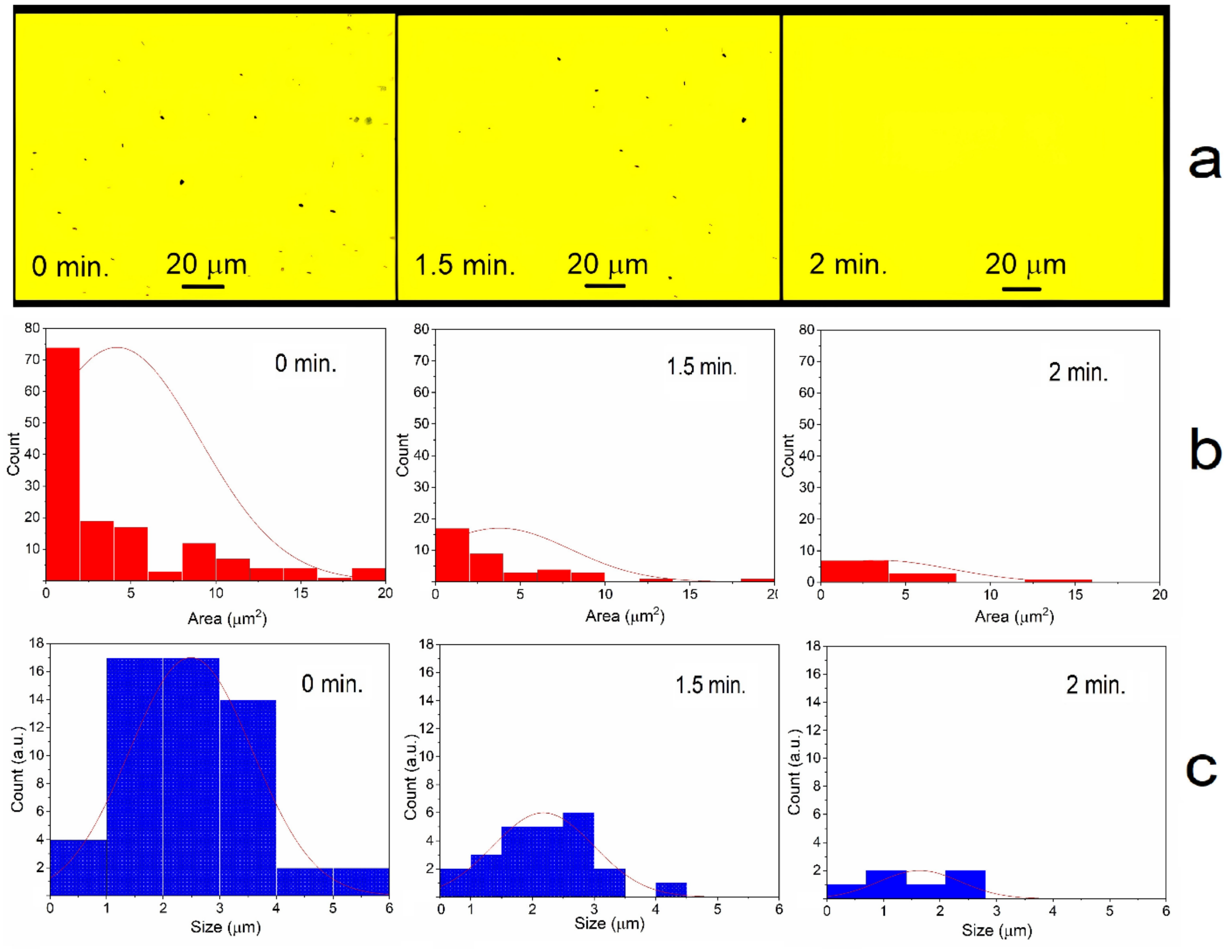

Figure S2. Micrographs of Tl microparticles in the volume of liquid (a), changes in their area (b) and size (c) over time. Magnification ×1000. Average particle sizes 2.5±0.1 μm (0 min.); 2.2±0.1 μm (1.5 min.) and 1.7±0.1 μm (2 min.).
